# Health system responses to population declines: call for papers

**DOI:** 10.2471/BLT.25.293300

**Published:** 2025-03-01

**Authors:** Jintana Jankhotkaew, Alana Officer, Ritu Sadana, Rapeepong Suphanchaimat, Angkana Lekagul, Walaiporn Patcharanarumol, Viroj Tangcharoensathien

**Affiliations:** a International Health Policy Foundation, Tiwanon Road, Nonthaburi, 11000, Thailand.; b Demographic Change and Healthy Ageing Unit, World Health Organization, Geneva, Switzerland.; c Ageing and Health Unit, World Health Organization, Geneva, Switzerland.

Globally, 63 countries reported that their populations peaked before 2024.^1^ The decline of working-age population, coupled with the rise in ageing population, can lead to increased demand for health care, social care, long-term care and pensions, in addition to diminished tax revenues and government fiscal capacity. 

Population declines result in workforce shortages, including health and care workforce, as in many countries, those who enter the health workforce cannot compensate for those who retire. Workforce shortage increases workload among health workers,^2^ compounds burnout and attrition and can compromise quality of care. High-income countries often resort to recruiting health workers from other countries, which affects health services in source countries.^3^ Such migration affects both source and destination countries by reducing the number of young, working-age individuals in origin countries while temporarily easing staffing shortages in destination countries.^4^

In light of the world’s rapidly ageing population, the United Nations General Assembly designated 2021–2030 as the Decade of Healthy Ageing, and Member States committed to implementing key actions to add healthy years to life.^5^ The Decade calls for well-designed health systems and supportive societal environments to promote healthy ageing, and a paradigm shift in attitudes, recognizing the potential and invaluable contributions of older adults. However, the 2021–2023 progress report on the Decade reveals that fewer than one third of countries have adequate resources to support key action areas.^6^ This gap underscores the need to translate political commitments into tangible actions – but doing so requires substantial implementation capacity.

Some older individuals live in vulnerable situations, with factors such as ethnicity, disability, mental illness, low socioeconomic status, limited education or poverty further compounding their vulnerability. In 2020, the World Health Organization (WHO) estimated that 142 million older individuals could not meet their basic needs, with women being more affected.^7^ Moreover, ageism is still prevalent. Ageism has multiple impacts on the health and well-being of older people.^8^ In some studies, more than half of older people surveyed experience loneliness and have lower scores of physical and mental health-related quality of life.^9^ This situation emphasizes the need for inclusive policies and community-driven interventions to address these issues.^10^

The *Bulletin of the World Health Organization* calls for papers on the multidimensional impacts of population decline and ageing. The *Bulletin* welcomes papers that explore multisectoral policy responses, including in health systems, aimed at ensuring sustainable social service infrastructure and health systems resilience through adequate workforce and financing to respond to these demographic transitions. Topics of interest include multisectoral policies that support social connection, combat ageism and enhance coordination of health and social care. Such policies include delivering support to caregivers and person-centred, integrated care and primary health services that are responsive to older people. Other priorities involve developing age-friendly environments, strengthening intergenerational solidarity and supporting independent living. Furthermore, appropriate economic and retirement policies that facilitate continuing employment or income-generation activities for older persons are needed.

Papers focusing on comprehensive economic and social policies to increase fertility rates, reduce poverty, maintain income, foster employment, improve gender equality and support early childhood development, are welcome. Additional topics of interest include effective multisectoral collaboration through a whole-of-government approach in response to population decline and ageing, along with strategies for health and care workforce and delivery modes of long-term care. Strategies include palliative care and the involvement of older persons and civil society in supporting older populations and technological adaptation. Papers could also explore the policy and practice of implementing advance directives to support dignified death and dying. Older people’s strengths and contributions to families, communities and society should be highlighted and acknowledged.

The *Bulletin* welcomes contributions from stakeholders, public health decision-makers, researchers and community representatives, especially from low- and middle-income countries.

The deadline for submissions is 16 June 2025. Manuscripts should be submitted in accordance with the *Bulletin’s* guidelines for contributors and the cover letter should mention this call for papers. This theme issue will be launched at the Prince Mahidol Award Conference in January 2026.
